# Survivorship care for cancer patients in primary versus secondary care: a systematic review

**DOI:** 10.1007/s11764-020-00911-w

**Published:** 2020-08-19

**Authors:** J. A. M. Vos, T. Wieldraaijer, H. C. P. M. van Weert, K. M. van Asselt

**Affiliations:** Department of General Practice, Amsterdam UMC, University of Amsterdam, Post-box 22660, 1100 DD Amsterdam, the Netherlands

**Keywords:** Cancer survivorship care, Primary care, Secondary care, Systematic review

## Abstract

**Background:**

Cancer survivorship care is traditionally performed in secondary care. Primary care is often involved in cancer management and could therefore play a more prominent role.

**Purpose:**

To assess outcomes of cancer survivorship care in primary versus secondary care.

**Methods:**

A systematic search of MEDLINE and EMBASE was performed. All original studies on cancer survivorship care in primary versus secondary care were included. A narrative synthesis was used for three distinctive outcomes: (1) clinical, (2) patient-reported, and (3) costs.

**Results:**

Sixteen studies were included: 7 randomized trials and 9 observational studies. Meta-analyses were not feasible due to heterogeneity. Most studies reported on solid tumors, like breast (*N* = 7) and colorectal cancers (*N* = 3). Clinical outcomes were reported by 10 studies, patient-reported by 11, and costs by 4. No important differences were found on clinical and patient-reported outcomes when comparing primary- with secondary-based care. Some differences were seen relating to the content and quality of survivorship care, such as guideline adherence and follow-up tests, but there was no favorite strategy. Survivorship care in primary care was associated with lower societal costs.

**Conclusions:**

Overall, cancer survivorship care in primary care had similar effects on clinical and patient-reported outcomes compared with secondary care, while resulting in lower costs.

**Implications for cancer survivors:**

Survivorship care in primary care seems feasible. However, since the design and outcomes of studies differed, conclusive evidence for the equivalence of survivorship care in primary care is still lacking. Ongoing studies will help provide better insights.

## Background

To date, the number of patients with incident cancer and cancer survivors is increasing, due to an aging population and the improvements in cancer screening, diagnosis, and treatment. Cancer survival has increased to over 60% between 2010 and 2020, as previously predicted by The Dutch Cancer Society [1]. In numerous countries worldwide, including the Netherlands, patients treated for cancer are initially included in a secondary care–based follow-up program, mainly focusing on the early detection of recurrences and treatment of symptoms caused by the cancer or its treatment. However, survivorship care for cancer encompasses not only detection of recurrences but also the attention to rehabilitation (psychological and social support, integration in society and secondary prevention) as addressed by the Institute of Medicine (IOM) back in 2006 [2].

Following completion of cancer treatment, many patients experience unmet needs and symptoms [3] and primary care is often involved in management of these needs and symptoms, especially for the older population with comorbidities [4–9]. A more general approach is therefore likely to be favorable to patient outcomes [10]. Traditional core values of primary care, such as the continuity and coordination of care, can lend themselves for the improvement of cancer survivorship, but the role of primary care may vary depending on context and setting [10, 11].

Several reviews have been published on alternative survivorship care strategies, such as GP-, PCP-, nurse-led, patient-initiated, and shared care [12–16]. However, none have focused exclusively on survivorship care by physicians working in primary care. The aim of this systematic review is to provide an overview of the outcomes of survivorship care in primary- compared with secondary-based care.

## Methods

### Study design and search strategy

In February 2020, a systematic search was performed in MEDLINE and EMBASE to identify original studies on cancer survivorship care. General terms for survivorship care, including follow-up and aftercare, were used. In addition to the MEDLINE and EMBASE search, reference checking was performed to identify possible other relevant publications. (See Appendix 1 for the search strategy.)

### Eligibility, selection, and data extraction

Original studies comparing cancer survivorship care in primary to secondary-based care were included. As health care systems differ around the globe, generalist professions providing primary- or community-based care, such as a general practitioner (GP), primary care physicians (PCPs), and family physicians (FPs), were included in this review. Studies reporting on patients of any age who were (curatively) treated for any type or stage of cancer were eligible. No restrictions were made on the type of outcomes. Economic evaluations of cancer survivorship care programs were also considered for inclusion. Studies on shared care and patient or physician preferences for survivorship care were excluded from this review.

All studies were screened on title and abstract by two independent researchers (JV and TW). Subsequently, complete texts were read to ensure inclusion criteria, and data were extracted. Data extraction was performed by one researcher (JV) based on a predefined data format. Disagreement between the two researchers on study selection and data extraction was resolved by discussion or, if necessary, by consulting a third independent researcher (KvA).

### Data analysis

As we intended a broad and conclusive review, no restrictions were made on the type of patient, outcomes, or methodology, which resulted in substantial heterogeneity of studies. Therefore, meta-analyses were not feasible and a narrative synthesis was used.

Outcomes were grouped into three distinctive categories: (1) clinical outcomes as measured by medical records (including survival, serious clinical events, and documented follow-up care), (2) patient-reported outcomes as measured by patient questionnaires and interviews (including quality of life, symptoms, patient satisfaction, and self-reported receipt of survivorship care), and (3) costs of survivorship care programs (including societal and patient costs).

### Quality assessment

A risk of bias analysis was performed for all included studies according to the designated quality assessment tools as advised by the Cochrane collaboration. The consort instrument was used for randomized clinical trials [17], and the ROBINS-I for (non-randomized) observational studies [18].

## Results

### Study selection

**Fig. 1:**
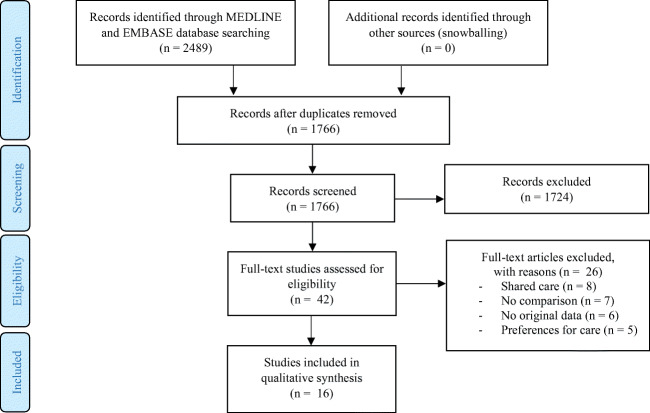
PRISMA flow diagram

The systematic search retrieved 1766 original studies (Fig. 1). Reference checking did not identify any additional studies. After title and abstract screening, full text of 42 studies was reviewed. Based on the predefined eligibility criteria, 16 studies were included in this review. Figure 1 illustrates the selection process.

### Quality assessment

Risk of bias assessment revealed low risk of bias in 10 studies, intermediate in 3, and high risk of bias in 3 out of 16 studies (see Appendix Table 5). Risk of bias was often inherent to the design of the study, including selection, misclassification, recall, and interviewer bias.

### Baseline characteristics

Table 1 shows the baseline characteristics of the included studies. Seven randomized controlled trials (RCTs) were included in this review [19–25]. Three studies of Grunfeld et al. were based on the same RCT, but reported on separate outcomes [20–22]. Other included studies were based on a type of observational study [26–34]. Most studies reported on patients with solid tumors, such as breast and colorectal cancers. The number of patients ranged from 98 in a retrospective cohort study [26] to 5009 in a quasi-experimental observational study [29]. Six studies reported on physicians working in primary care, of which two studies did not further specify the provider type [31, 32]. The length of follow-up ranged from 1 to 15 years.

**Table 1:** Baseline characteristics of the included studies; (a) randomized controlled trials and (b) observational studies

Author, year, country	Participants	Intervention	Time since diagnosis	Outcome	Baseline differences
(a) Randomized controlled trials					
Augestad et al., 2013, Norway[19]	Patients < 75 years curatively treated for colon cancer, Dukes’ stage A, B, or C (*N* = 110). Transfer of care approximately 3–4 weeks after surgery.	GP- (*N* = 55) versus surgeon-led survivorship care (*N* = 55).	2 years	Clinical, patient-reported and costs	No significant differences.
Grunfeld et al., 1996, 1999 and 1999, UK[20–22]	Women curatively treated for early-stage breast cancer I–III (*N* = 296). Transfer of care after mean 3.4 months (SD 1.8).	GP- (*N* = 148) versus hospital-based survivorship care (*N* = 148).	18 months	Clinical, patient-reported and costs	Patients in the hospital-group were older (59.0 versus 55.6 years) and had more stage I tumors (50.6% versus 40.5%).
Grunfeld et al., 2006, UK[23]	Woman curatively treated for early-stage breast cancer I–III (*N* = 968). Transfer of care between 9 and 15 months after curative treatment.	FP- (*N* = 483) versus specialist-led survivorship care (*N* = 485).	5 years	Clinical and patient-reported	No significant differences.
Murchie et al., 2010, UK[24]	Patients curatively treated for primary cutaneous melanoma (*N* = 142). Transfer of care after median 49 months (IQR 19–76).	GP- (*N* = 53) versus hospital-based survivorship care (*N* = 89).	1 year	Clinical and patient-reported	Patients in the GP-group lived further away to the hospital (27.6 miles (18.9–32.3) versus 10.1 (2.3–25.9)).
Wattchow et al., 2006, Australia[25]	Patients curatively treated for colon cancer, Dukes’ stage A, B, or C (*N* = 203). Transfer of care approximately 4–6 weeks after surgery or chemotherapy.	GP- (*N* = 97) versus surgeon-led survivorship care (*N* = 106).	2 years	Clinical and patient-reported	A trend towards higher levels of education was seen for patients in the surgeon-group (postsecondary school 22.5% versus 8.5%).
(b) Observational studies					
Baena-Canada et al., 2013, Spain[26]	Woman curatively treated for early stage breast cancer 0–III (*N* = 98). Transfer of care 5 years after primary treatment.	Primary care- (*N* = 60) versus hospital-based survivorship care (*N* = 38).	10 years	Clinical, patient-reported and costs	Patients in the primary care-group were older (60 versus 38 year, *p* = 0.002) and received less chemotherapy (62% versus 87%, *p* = 0.001).
Haggstrom et al., 2009, USA[27]	Colorectal cancer survivors (*N* = 416). Transfer of care unknown.	Comparison of physician specialty most often seen; no physician (*N* = 113), PCP (*N* = 50), oncologist (*N* = 183), surgeon (*N* = 29) or gastroenterologist (*N* = 41).	1 year	Patient-reported	Patients were more inclined to receive care by a specialist if; stage III or IV disease (*p* = 0.03) and fewer comorbid medical conditions (*p* = 0.012).
Maly et al., 2013, USA[28]	Low-income women aged ≥ 18 years diagnosed with breast cancer stage 0–III (*N* = 579). Transfer of care unknown.	Comparison of physician specialty most often seen; PCP only (*N* = 40), specialist only (*N* = 100) or shared care (*N* = 439).	36 months	Patient-reported	No baseline analyses.
Mittmann et al., 2018, Canada[29]	Woman curatively treated for any stage of breast cancer (*N* = 5009). Transfer of care unknown.	PCP- (*N* = 2685) versus traditional cancer clinic–based survivorship care (*N* = 2324).	25 months	Clinical and costs	No differences.
Parry et al., 2015, UK[30]	Patients diagnosed with stable stage A0 chronic lymphocytic leukemia (*N* = 246). Transfer of care unknown (after second outpatient visit).	GP- (*N* = 105) versus hospital-based survivorship care (*N* = 141).	Median 66 months (IQR 49–94) in GP-group	Clinical	Patients in the GP-group were older (median age 71 versus 68, *p* = 0.02) and white cell count at diagnosis was higher (median 13.2 versus 10.4, *p* = 0.018).
Railton et al., 2015, Canada[31]	Women aged ≥ 18 years treated for stage I–III invasive breast cancer (*N* = 240). Transfer of care after median 11.3 months (range 1.8–42.0).	PCP community- (*N* = 171) versus cancer center-based survivorship care (*N* = 69).	From 12 ≥ 48 months	Patient-reported	Patients in PCP-group were older (59.1% ≥ 50 years versus 39.1%, *p* = 0.005) and had more stage I disease stage (50.9% versus 37.7%, *p* = 0.01).
Risendal et al., 2016, USA[32]	Woman curatively treated for breast cancer (*N* = 298). Transfer of care unknown.	PCP- (*N* = 94) versus oncologist-led survivorship care (*N* = 204).	Average 6.7 years (SD = 0.98)	Patient-reported	Patients in the PCP-group were older (26.6% ≥ 65 years versus 8.8%, *p* < 0.01) and more frequently had a lapse in insurance (22.6% versus 9.6%, *p* < 0.01) and in situ disease (33.0% versus 12.8%, *p* < 0.01).
Samawi et al., 2018, Canada[33]	Patients aged ≥ 18 years who received at least one cycle of adjuvant chemotherapy after curative resection of pancreatic adenocarcinoma (*N* = 147). Transfer of care unknown.	PCP- (*N* = 50) versus cancer center-based survivorship care (*N* = 97).	15 years	Clinical	Patients in the PCP-group had more T1/T2 tumors (38.0% versus 21.6%, *p* = 0.03).
Peixoto et al., 2014, Canada[34]	Patient aged ≥ 18 years treated for non-metastatic gastroesophageal cancer with curative-intent (*N* = 292). Transfer of care unknown.	Comparison of 4 survivorship care strategies; discharge to GP (*N* = 89), care by oncologist with clinical assessments (*N* = 18), specialist care with laboratory investigations (*N* = 32), or specialist care with imaging or endoscopy (*N* = 153).	3 years	Clinical	Patients were more inclined to receive care by a specialist if; gastroesophageal junction or gastric tumors (*p* = 0.001), primary lesions involving the distal esophagus (p = 0.001), specific histological subtypes (*p* = 0.008) and definitive chemoradiotherapy (*p* = 0.001).

*GP* general practitioner, *FP* family physician, *PCP* primary care physician

### Clinical outcomes

Ten studies reported on clinical outcomes (see Table 2). No important differences were seen in survival between follow-up strategies after 3 up to 15 years of follow-up [30, 33, 34]. Follow-up in secondary care was associated with shorter relapse-free survival (RFS) and higher likelihood of receiving palliative treatment with chemotherapy (58% versus 34%, *p* = 0.03) in pancreatic cancer patients in a cohort study, in part because patients in secondary care had more advanced primary tumors [33]. Eight studies examined the occurrence of serious clinical events. No differences were seen relating to the number (and time of diagnosis) of recurrences and metastases [19, 20, 23–26], deaths [23, 25, 29], or other clinical events [23, 24, 26] between primary and secondary care–based follow-up.

**Table 2:** Clinical outcomes of cancer survivorship care including (a) survival, (b) serious clinical events, and (c) documented follow-up care

Clinical	Ref.	Result
(a) Survival		
Overall survival (OS)	[30]	69.5% (GP) versus 68.6% (hospital), *p* = 0.888.
	[33]	In multivariate analyses a HR of 0.81 (PCP), CI 0.49–1.35, *p* = 0.43.
	[34]	Reported as a figure, *p* = 0.34, non-significance remained in multivariate analyses.
Relapse-free survival (RFS)	[30]	83.0% (GP) versus 78.1% (hospital) remained asymptomatic. 17.0% (GP) versus 21.9% (hospital) needed treatment, *p* = 0.424. No differences were seen relating to the time to first treatment (*p* = 0.188).
	[33]	Patients in the PCP-group had longer RFS; in multivariate analyses a HR of 0.62 (PCP), CI 0.39–0.98, *p* = 0.041. Patients in the PCP-group were less likely to receive palliative chemotherapy for their relapse (34% versus 58%, *p* = 0.03).
	[34]	Reported as a figure, *p* = 0.59, non-significance remained in multivariate analyses.
(b) Serious clinical events		
Recurrences and metastases	[19]	6 recurrences (GP) versus 8 (hospital), mean time to diagnosis was 35 days (GP) versus 45 days (hospital), *p* = 0.46.
	[20]	6.8% recurrences (GP) versus 10.8% (hospital), median time to diagnosis 22 days (GP) versus 21 days (hospital), median difference in time to diagnosis 1.5 days (range − 13 to 22).
	[23]	11.2% recurrences (FP) versus 13.2% (specialist), difference 2.02%, CI − 2.13–6.16%.
	[24]	In both groups 1 diagnosis of recurrent melanoma.
	[25]	Recurrence rate of 7.1 per 1000 months (GP) versus 8.0 (surgeon), *p* = 0.92, median time to detection was 9.5 (GP) versus 8.0 months (surgeon), *p* = 0.76.
	[26]	1.6% metastases (primary) versus 0% (hospital), *p* = 0.74.
Deaths	[23]	6.0% death of any cause (FP) versus 6.2% (specialist), difference 0.18%, CI − 2.90–3.26.
	[25]	Death rate of 6.6 per 1000 months (GP) versus 5.4 (surgeon), *p* = 0.67, median time to death 31 (GP) versus 20 months (surgeon), *p* = 0.69.
	[29]	18.6% death of any cause (PCP) versus 20.3% (specialist), *p* = 0.7.
Other clinical events	[23]	Recurrence-related events (such as hypercalcemia or fracture); 3.5% (FP) versus 3.7% (specialist), difference 0.19%, CI − 2.26–2.65%.
	[24]	New primary tumors (1 new primary in both groups).
	[26]	New primary tumor (5% (primary) versus 10.4% (hospital), *p* = 0.67), associated diseases (46.7% (primary) versus 60.5% (hospital), *p* = 0.21) and treatment effects (22.3% (primary) versus 21.1% (hospital), *p* = 0.79).
(c) Documented follow-up care		
Adherence to medical guidelines and follow-up tests	[24]	Patients who visited a GP were more likely to be seen according to guideline (98.1% versus 80.9%, *p* = 0.020).
	[25]	Patients in the GP-group were more likely to visit their physician (1.27 times per quarter versus 0.84 times) and to have one or more FOBTS (rate ratio 2.4, CI 1.4–4.4, *p* = 0.003). Patients in the surgeon group were more likely to have ultrasounds (rate ratio 0.5, CI 0.3–1.0, *p* = 0.040) and colonoscopies (rate ratio 0.7, CI 0.5–1.0, *p* = 0.027). No differences were seen relating to other surveillance tests, including CEA, X-ray and CT-scan.

*GP* general practitioner, *FP* family physician, *PCP* primary care physician

Documented follow-up care, as measured by adherence to medical guidelines and follow-up tests, was assessed by two RCT’s. Murchie et al. [24] found that 98.1% of patients in primary-based care were seen according to guidelines versus 80.9% of patients in secondary-based care (*p* = 0.020). In the second study, patients in primary care were more likely to have one or more fecal blood tests (rate ratio 2.4, CI 1.4–4.44, *p* = 0.003), whereas patients in secondary care were more likely to have ultrasounds and colonoscopies, although it remained unclear whether or not this was done in accordance with follow-up guidelines [25].

### Patient-reported outcomes

Eleven studies, including all six RCTs, measured patient-reported outcomes (see Table 3). After adjustment for clinical and pathological covariates, no differences were seen in overall quality of life (QoL) and anxiety and depression between survivorship care strategies [19, 20, 23–26]. One observational study examined other bothersome symptoms, showing less fatigue among breast cancer patients in primary care (62.0% versus 81.1%, *p* = 0.005) [31].

**Table 3:** Patient-reported outcomes of cancer survivorship care including (a) quality of life, (b) symptoms, (c) patient satisfaction and perception of care, and (d) self-reported receipt of survivorship care

Patient-reported	Ref.	Method	Result
(a) Quality of life (QoL)			
	[19]	EORTC QLQ C-30, EuroQol-5D, and EQ VAS at baseline up to 24 months.	No differences in overall QoL, effects on subscales in favor of GP in role functioning (mean difference − 5.1 (CI − 9.7 to − 0.5), *p* = 0.02), emotional functioning (− 3.7 (CI − 6.8 to − 0.6), *p* = 0.01) and pain (4.5 (CI 0.8–8.2), *p* = 0.01).
	[20]	SF-36 and EORTC QLQ C-30 at baseline, mid- and end-of-trial.	No differences on any subscale.
	[23]	SF-36 at baseline up to 60 months.	No differences on any subscale.
	[24]	SF-36 at baseline and 12 months.	No differences on any subscale.
	[25]	SF-12 PCS and MCS scores at baseline, 12 and 24 months.	No differences on any subscale.
	[26]	SF-36 (administered once more than 5 years after treatment).	No differences on any subscale after adjustment for age and chemotherapy.
(b) Symptoms			
Anxiety and depression	[20]	HADS at baseline, mid- and end-of-trial.	Anxiety scale difference 0.4 (CI − 0.3 to 1.2), depression scale difference 0.4 (CI − 0.2, to 1.1).
	[23]	HADS at baseline up to 60 months.	Reported as a figure; no differences.
	[24]	HADS at baseline and 12 months.	8 patients were diagnosed as anxious (GP) versus 13 (hospital) (*p* = 0.868), 3 patients as depressed (GP) versus 5 (hospital) (*p* = 0.912).
	[25]	HADS at baseline, 12- and 24 months.	Median anxiety score 4.0, IQR 5.0 (GP) versus 5.0, IQR 4.5 (surgeon) (*p* = 0.106), median depression score 4.0, IQR 5.0 (GP) versus 3.0, IQR 4.0 (hospital) (*p* = 0.796).
Other bothersome symptoms	[31]	One-time structured telephone interview.	Patient who visited a PCP had less fatigue (62.0% versus 81.1%, *p* = 0.005). No differences for other symptoms (arthralgias, hot flashes, memory loss, vaginal dryness, insomnia, paresthesias and depression).
(c) Patient satisfaction and perception of care			
	[22]	Adapted satisfaction questionnaire at baseline, mid-, and end of trial (Cronbach’s alpha = 0.70).	Patients who visited a GP had greater satisfaction on 9 out of 15 aspects (relating to service delivery, consultation and continuity of care).
	[24]	Patient questionnaire at baseline and 12 months (Cronbach’s Alpha = 0.70).	Patient who visited a GP had greater satisfaction on 6 out of 15 aspects (relating to service delivery, consultation and continuity of care), the mean score was 26.4, CI 24.9–27.9 (GP), versus 33.5, CI 32.5–34.4 (hospital), the change in mean summary score was −5.96, CI − 8.09–3.89 (GP), versus 0.29, CI − 1.49–2.08 (hospital), indicating higher satisfaction with GP (*p* < 0.001).
	[25]	PSVQ at 24 months (previously validated).	No differences on any subscale.
	[26]	Questionnaire (administered once, more than 5 years after treatment, Cronbach’s Alpha = 0.88).	Patients who visited a specialist had greater satisfaction on all 6 dimensions; health care attention (*p* = 0.001), attention by medical (*p* = 0.006) and nursing personnel (*p* = 0.016), recommendation of service (*p* = 0.019), information provision (*p* = 0.003) and respect/friendliness (*p* = 0.008).
	[27]	Adapted patient questionnaire on perceptions of follow-up 2–5 years after diagnosis.	No differences in communication, coordination, nursing care, office staff and follow-up rating; non-significance remained in multivariate regression.
	[32]	Computer-aided telephone interview on perception of follow-up.	Women who visited an oncologist reported a marginally higher degree of care coordination (81.9% versus 73.1%, OR 1.8, CI 1.0–3.5).
(d) Self-reported receipt of survivorship care			
Adherence to medical guidelines and follow-up tests	[27]	Patient questionnaire on visits, tests and examinations 2–5 years after diagnosis.	The number of visits in the past year varied by physician specialty (*p* < 0.001). Patients in the PCP-group were less likely to see a doctor for “follow-up medical tests” (68% versus 89%, *p* < 0.001) and more likely to receive a physical examination (58% versus 36%, *p* = 0.004). PCPs more often helped with lifestyle improvements (83% versus 63%, *p* = 0.015) and discussed diet (70% versus 48%, *p* = 0.005).
	[28]	Patient survey on receipt of preventive care at baseline, 6, 18 and 36 months after diagnosis.	Patients who visited a PCP only were more like to receive a Pap smear (AOR 2.90, CI 1.05–8.04, *p* = 0.040) and colonoscopy (AOR 2.99, CI 1.5–8.51, *p* = 0.041). No differences were seen in receipt of mammogram (*p* = 0.109).
	[31]	One-time structured telephone interview on receipt of follow-up.	Patients who visited a PCP had fewer clinical examinations (85.6% versus 95.7%, *p* = 0.04), no differences were seen in accessing physician for examination, receipt of mammograms, having an endocrine therapy plan, psychosocial and sexual health, lifestyle management or need for assistance with follow-up goals.
	[32]	Computer-aided telephone interview on receipt of follow-up.	Patients who visited a PCP were less likely to receive a clinical breast exam (79.6% versus 90.2%, OR 2.5, CI 1.2–5.5). No differences were seen in receipt of mammogram, X-ray, scans, physical exam or education about breast self-exam. Women who visited an oncologist reported more tumor marker (OR 3.0, CI 1.5–5.8) and other blood tests (OR 2.0, CI 1.1–3.5).

*GP* general practitioner, *PCP* primary care physician

High levels of patient satisfaction and perception of care were found for survivorship care in both primary- and secondary-based care [22, 24–27, 32]. Using an adapted validated questionnaire, higher levels of patient satisfaction were found in primary care–based groups in two RCTs (9 out of 15 aspects by Grunfeld et al. [22] and 6 out of 15 by Murchie et al. [24]). In contrast, a questionnaire administered in an observational study [26] showed greater satisfaction in all 6 dimensions for breast cancer patients in secondary-based care (*p* < 0.05).

Five observational studies examined self-reported receipt of survivorship care by means of questionnaires and interviews. Disparate results were seen among primary- and secondary-based care, but there was no evidence for a more favorable strategy based on these results. Two studies showed a lower adherence to recommended periodic clinical examinations for breast cancer patients by physicians working in primary care (approximately 80% versus 90% in secondary care, *p* < 0.05) [31, 32]. In another study, patients in primary care were more likely to receive examination as is recommended by national guidelines (58% versus 36%, *p* = 0.004) [27]. No differences were seen in patient self-reported mammogram frequency [28, 31, 32]. Maly et al. [28] found a higher uptake of preventive tests, including Pap smear (AOR 2.90, CI 1.05–8.04, *p* = 0.040) and colonoscopy (AOR 2.99, CI 1.5–8.51, *p* = 0.041), among underserved breast cancer patients in primary care. Physicians in primary care helped more often with lifestyle improvements for colorectal cancer patients [27], but this was not the case among breast cancer patients [31].

### Costs

Survivorship care in primary care was associated with lower societal and patient costs in all four studies that performed cost analyses (see Table 4) [19, 21, 26, 29]. The main cost driver in all studies was the mean cost per visit, including organizational and physician costs.

**Table 4:** Costs of cancer survivorship care including (a) societal costs and (b) patient costs

Costs	Ref.	Method	Result
(a) Societal costs			
	[19]	Cost- and utilization questionnaire filled in by patients at baseline up to 24 months (including visits, tests and events such as metastases).	Mean cost of follow-up per patient per follow-up cycle was £292 (GP) versus £351 (surgeon), *p* = 0.02. Mean societal cost per patient for 24 months follow-up was £8233, range £7904 to £8619 (GP), versus £9889, range £9569 to £10,194 (surgeon), mean difference in favor of GP £1656, *p* < 0.001.
	[21]	Record-of-visit form filled in by doctors at baseline up to 18 months (including visits and tests).	Mean total cost per patient for 18 months follow-up was £64.7, range £5.8–301.9 (GP) versus £195.1, range £62.0–737.4 (surgeon), mean difference £130.4 (range £–149.1;–111.6) in favor of GP, *p* < 0.001. Difference mainly due to mean cost of visit (mean cost £40.9, range £5.8–143.8 (GP), versus £174.1, range 62.0–558.0 (surgeon)).
	[26]	Direct costs based on a single national database (Consejería de Salud, Junta de Andalucía) (including visits and tests).	Total cost of follow-up per patient per year was mean €112.86, SD 77.54 (primary care), versus mean €184.61, SD 85.87 (hospital), *p* = 0.0001. Difference mainly due to costs per visit (mean €17.46, SD 8.62 (primary), versus mean €60.32, SD 21.19 (hospital), *p* < 0.001).
	[29]	Direct costs based on multiple national databases (including visits, tests, medication and events such as hospitalization).	Mean annual total cost per survivor was CAD $6575, CI $5563 to $7587 (PCP) versus $10,832, CI $9947 to $11,717 (specialist), resulting in $4257, CI $2928 to $5587, lower annual cost (39.3% reduction) per survivor in the PCP group. A 22.1% reduction in overall median annual costs ($2261 versus $2903) was seen. Main cost drivers included hospitalization, physician visits, medications, and home care. The PCP group had lower mean annual costs for same-day surgery, cancer clinic visits, physician visits, medications, long-term care, and home care.
(b) Patient costs			
	[19]	Cost- and utilization questionnaire filled in by patients at baseline up to 24 months (including travel, out-of-pocket expenses and work loss).	More patients had expenses relating to travel in the hospital-group (£156.9 versus £76.7, p < 0.001). No differences were seen relating to out-of-pocket expenses (p = 0.10) or work loss (*p* = 0.45).
	[21]	Cost-questionnaire filled in by patients at baseline up to 18 months (including travel, out-of-pocket expenses, work loss, child support and spent time for an appointment).	Patients in the GP-group were more frequently in paid employment (47.8% versus 31.0%, *p* = 0.023), walked to their appointment (32.4% versus 1.6%, p = 0.000), spent less time getting to their appointment (13.1 min (SD 8.3) versus 26.7 (SD 15.9)), spent more time during the appointment (52.6 min (SD 22.1) versus 82.2 (SD 31.8)). Patients in the hospital-group took more time off work (61.1% versus 32.3%, *p* = 0.006) and had more out-of-pocket expenses (including parking fare, 11.0% versus 2.4%, *p* = 0.008). No differences were seen in the proportion of patients losing wages (*p* = 0.24) or in need of child care (*p* = 0.06).

*GP* general practitioner, *PCP* primary care physician

## Discussion

In this review, similar effects on clinical and patient-reported outcomes were seen for survivorship care in primary- compared with secondary-based care. Although the evidence should be interpreted with caution, survivorship care in primary care seems feasible and results in lower costs.

### Comparison with existing literature

A recent Cochrane review found little to no effects on pre-defined outcomes for RCTs comparing non-specialist (e.g., PCP-led, nurse-led, patient-initiated, and shared care) to specialist-led follow-up [12]. The certainty of evidence was generally low due to the limited amount of RCTs. Similarly to the Cochrane review, this review found no important differences in survivorship care between primary and secondary care relating to clinical (survival and recurrences) and patient-reported outcomes (quality of life and symptoms).

This review has identified additional outcomes in comparison with the Cochrane review relating to the content and quality of survivorship care. The content of survivorship care is examined by both documented follow-up care and self-reported receipt of survivorship care. Some differences were seen in these outcomes, especially relating to the adherence to guidelines and follow-up tests, but the results showed no favorite strategy. It remains unclear whether or not these differences may affect other outcomes, such as detection of recurrences and survival. Showing differences in these types of outcomes requires great numbers of patients and considerable follow-up time among often older patients with comorbidities, making this a challenging undertaking.

This review has examined patient’s perceptions and satisfaction with care as indicators for the quality of survivorship care. High levels of quality of care were found for survivorship care in both primary- and secondary-based care. Two out of three RCTs showed higher levels of patient satisfaction with primary-based care, illustrating its feasibility [22, 24]. The aggregation of these results provides us with the indication that survivorship care in primary care is similar to care by a specialist. Moreover, survivorship care in primary care led to lower costs in all studies that performed cost-analyses.

### Strengths and limitations

Our review provides additional evidence to previous literature by focusing exclusively on survivorship care by physicians working in primary care and by including non-randomized studies in the results. By performing a non-restrictive search and selection strategy, two additional outcomes relating to the content and quality of survivorship care have been identified in comparison with the recent Cochrane review. The search strategy, including reference checking, provides a sensitive search result.

There are some limitations that need to be addressed. Inherent to the design of some studies, differences were seen in baseline characteristics. Older patients and patients with prognostic better disease stage were sometimes more likely to receive follow-up in primary care [26, 27, 30–34]. Despite adjusting for covariates, these differences might have influenced outcomes. Due to the substantial heterogeneity in outcomes and methodology, no data could be pooled for meta-analyses, hampering the interpretation of results. However, using a narrative synthesis, no important differences were seen relating to clinical and patient-reported outcomes. These results are in line with previous reviews [12–16].

### Implications for future practice and research

As the number of cancer survivors is rapidly increasing and resources are limited [12–16], alternative survivorship care strategies for the hospital-based survivorship care are deemed desirable. This review showed that cancer survivorship care in primary care seems feasible and worthwhile to consider. However, the role and capacity of physicians in primary care can vary depending on context and setting [10, 11]. Most studies were performed in the UK and Canada in which physicians work as gate-keepers to secondary health care services. In these countries, a publicly funded universal health care system is in place. Other studies were performed in countries such as the US and Spain in which the health care system is both publicly and privately funded, and the role of primary care could be less distinguished. The randomized trials that could be identified, were limited to countries with a universal health care system, so further research is warranted to evaluate whether the results of these trials are also applicable to other health care systems. Furthermore, both clinical and patient-reported outcomes might change over time and could be affected by the length of follow-up. Therefore, to assess durable effects of survivorship care, greater number of patients and considerable follow-up time in these trials would be preferable. Moreover, the impact on the work-load for primary care physicians needs to be evaluated in case of growing numbers of patients in primary care–based cancer survivorship care.

## Conclusion

This review presents a comprehensive overview of survivorship care in primary care. To our opinion, this review has underlined the feasibility of survivorship care in primary care or possibility of some form of cooperative care. However, delivering high-quality survivorship care will also put restraints on primary care. This requires not only sufficient funding but also investments in organization and staff. Further studies with adequate designs are needed.

## Figures and Tables

**Table 5:**
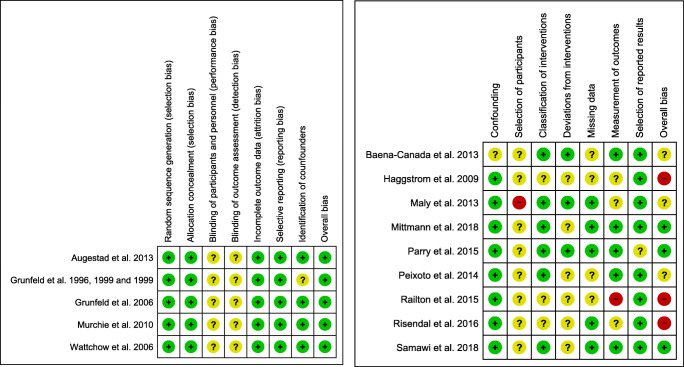
Risk of bias assessment

## Appendix 1. Search strategy

The following search strategy was used in MEDLINE. The search strategy was developed in collaboration with a medical librarian (FvE) of the Amsterdam UMC, location AMC. A total of 1766 studies were found from inception up to the 22nd of February of 2020.

**Table:**

History				
Search	Add to builder	Query	Items found	Time
#5	Add	Search ((((#1) AND #2) AND #3) AND #4 Sort by: Publication Date	1385	09:49:37
#4	Add	Search (“Cohort Studies”[Mesh] OR “Aftercare”[Mesh] OR follow-up*[tiab] OR followup*[tiab] OR aftercare[tiab] OR after-care[tiab] OR surveillance*[tiab] OR post-operat*[tiab] OR postoperat*[tiab] OR post-surg*[tiab] OR postsurg*[tiab] OR post-treatment*[tiab] OR posttreatment*[tiab] OR checkup*[tiab OR check up*[tiab]) Sort by: PublicationDate	3096777	09:49:12
#3	Add	Search (“Secondary Care”[Mesh] OR “Secondary Prevention”[Mesh] OR dermatologist*[tiab] OR oncologist*[tiab] OR surgeon*[tiab] OR gastroenterologist*[tiab] OR urologist*[tiab] OR specialistled[tiab] OR secondary care[tiab] OR secondary healthcare[tiab] OR secondary health care[tiab] OR hospital care[tiab] OR hospital follow up[tiab] OR hospital-based follow-up[tiab] OR cancer center*[tiab] OR specialist care[tiab]) Sort by: PublicationDate	290700	09:48:59
#2	Add	Search (“Family Practice”[Mesh] OR “Primary Health Care”[Mesh] OR “Physicians, Primary Care”[Mesh] OR “Physicians, Family”[Mesh] OR “General Practitioners”[Mesh] OR “General Practice”[Mesh] OR family practi*[tiab] OR family care*[tiab] OR family healthcare*[tiab] OR family health care*[tiab] OR primary care*[tiab] OR primary healthcare*[tiab] OR primary health care*[tiab] OR general practi*[tiab] OR GP[tiab] OR GPs[tiab] OR PCP[tiab] OR PCPs[tiab] OR family doctor*[tiab] OR family physician*[tiab]) Sort by: PublicationDate	404255	09:48:49
#1	Add	Search (“Neoplasms”[Mesh] OR “Cancer Survivors”[Mesh] OR neoplasms*[tiab] OR cancer*[tiab] OR tumor*[tiab] OR tumour*[tiab] OR malignan*[tiab]) Sort by: PublicationDate	4174383	09:48:35

The search was subsequently translated and adapted for use in EMBASE. A total of 1104 studies were found in EMBASE (classic)

**Table:**

#	Searches	Results
1	exp Neoplasms/ or Cancer Survivors/ or (neoplasm* or cancer* or tumor* or tumor* or malignan*).ti,ab,kw.	5,705,657
2	general practice/ or general practitioner/ or exp. primary health care/ or (family practi* or family care* or family healthcare* or family health care* or primary care* or primary healthcare* or primary health care* or general practi* or GP or GPs or PCP or PCPs or family doctor* or family physician*).ti,ab,kw.	469,424
3	exp secondary health care/ or (dermatologist* or oncologist* or surgeon* or gastroenterologist* or urologist* or specialist led or secondary care or secondary healthcare or secondary health care or hospital care or cancer center* or specialist care or hospital follow up or hospital-based follow-up).ti,ab,kw.	45,998
4	cohort analysis/ or exp. aftercare/ or (follow-up* or followup* or aftercare or after-care or surveillance* or post-operat* or postoperat* or post-surg* or postsurg* or post-treatment* or posttreatment* or checkup* or check up*).ti,ab,kw.	3,217,165
5	1 and 2 and 3 and 4	1965
6	limit 5 to conference abstract status	932
7	5 not 6	1104

After removal of duplicates, a total of 1766 studies were screened on title and abstract
